# Transient opening of trimeric prefusion RSV F proteins

**DOI:** 10.1038/s41467-019-09807-5

**Published:** 2019-05-08

**Authors:** Morgan S. A. Gilman, Polina Furmanova-Hollenstein, Gabriel Pascual, Angélique B. van ‘t Wout, Johannes P. M. Langedijk, Jason S. McLellan

**Affiliations:** 1 Department of Biochemistry and Cell Biology, Geisel School of Medicine at Dartmouth, Hanover, NH 03755 USA; 2 Department of Molecular Biosciences, University of Texas at Austin, Austin, TX 78712 USA; 3 Janssen Infectious Diseases and Vaccines, Leiden, CN 2333 The Netherlands; 4 Janssen Immunosciences, World Without Disease Accelerator, San Diego, CA 92121 USA; 5 Janssen Prevention Center, Janssen Vaccines & Prevention B.V, Leiden, CN 2333 The Netherlands; 6 AlphaBiomics, London, SW4 0PA United Kingdom

**Keywords:** Viral membrane fusion, X-ray crystallography, Viral infection

## Abstract

The respiratory syncytial virus (RSV) F glycoprotein is a class I fusion protein that mediates viral entry and is a major target of neutralizing antibodies. Structures of prefusion forms of RSV F, as well as other class I fusion proteins, have revealed compact trimeric arrangements, yet whether these trimeric forms can transiently open remains unknown. Here, we perform structural and biochemical studies on a recently isolated antibody, CR9501, and demonstrate that it enhances the opening of prefusion-stabilized RSV F trimers. The 3.3 Å crystal structure of monomeric RSV F bound to CR9501, combined with analysis of over 25 previously determined RSV F structures, reveals a breathing motion of the prefusion conformation. We also demonstrate that full-length RSV F trimers transiently open and dissociate on the cell surface. Collectively, these findings have implications for the function of class I fusion proteins, as well as antibody prophylaxis and vaccine development for RSV.

## Abstract (web summary)

The respiratory syncytial virus (RSV) F glycoprotein forms a trimeric complex and mediates viral entry. Using structures of RSV F in complex with antibodies, Gilman et al. here show a breathing motion of the prefusion conformation of F, resulting in transient opening of the trimeric complex in solution and on the cell surface.

## Introduction

Respiratory syncytial virus (RSV) is an enveloped RNA virus that is a member of the recently established *Pneumoviridae* family^1^. Upper respiratory tract infections due to RSV reoccur multiple times throughout life, but rarely lead to severe complications in healthy adults. However, RSV infections in infants, the elderly, and the immunocompromised can lead to bronchiolitis or pneumonia, which may result in hospitalization or even death. These complications are a substantial cause of infant mortality worldwide, resulting in approximately 60,000 in-hospital deaths annually in children under the age of 5^2^. Although mortality due to RSV infections is rare in developed countries, the economic burden associated with RSV is substantial. In the United States, for example, it is estimated that 652 million dollars were spent in 2000 alone on RSV-related medical costs^3^. Although prophylaxis with the monoclonal antibody palivizumab reduces the risk of hospitalization associated with RSV, it must be delivered intravenously multiple times per RSV season and has modest efficacy, preventing its use in developing regions^4,5^.

Two glycoproteins are present on the surface of RSV—the attachment protein (RSV G) and the fusion protein (RSV F). RSV G enhances pathogenicity in vivo and facilitates attachment to host cells^6^, but is not absolutely required for infection^7,8^. RSV F is a class I fusion protein that mediates fusion of the viral membrane with a host-cell membrane, a process that poses an otherwise insurmountable energetic barrier to viral entry. Initially, the F protein exists in a metastable prefusion conformation that is anchored in the viral membrane by a transmembrane domain. In this conformation the hydrophobic fusion peptide is buried within the central cavity of the protein^9^. An unknown event causes prefusion F to undergo a dramatic conformational change, resulting in refolding of five distinct structural elements adjacent to the fusion peptide into a single alpha helix and insertion of the fusion peptide into the host-cell membrane. In this state, called the pre-hairpin intermediate, two heptad repeats (HRA and HRB) are positioned on opposing ends of the F molecule. The collapse of these heptad repeats into a stable six-helix bundle is an energetically favorable conformational change that is coupled to the fusion of the two lipid bilayers^10,11^. Antibodies that bind to F can interrupt the fusion process, thereby preventing viral entry and reducing the severity of RSV-related disease^5,12,13^. Thus, there is great interest in the isolation of RSV F-directed antibodies with neutralizing activity that could serve as the basis for improved therapeutics.

Mapping the antigenic topology of RSV F previously relied upon competition assays and isolation of viral escape mutations^14–17^, but recent efforts have expanded upon these maps and placed them in the context of the high-resolution structures for both pre- and postfusion F^9–11,18^. Among the first antigenic sites to be defined was antigenic site II, which is composed of a helix-turn-helix motif that spans residues 253–278 and is the target of palivizumab^19–21^. This motif is present on both the pre- and postfusion conformations of F, and antibodies that recognize site II generally bind equally well to both^9,22^. Recently, it has been established that antibodies that specifically recognize the prefusion conformation of F are in general more potently neutralizing than antibodies that also bind to postfusion F^23^. The first prefusion-specific antibodies that were isolated recognized the apex of the prefusion F trimer and neutralized RSV over tenfold more potently than palivizumab^9,24^. This region was named antigenic site Ø and was later shown to be a primary target of RSV-neutralizing activity in human sera^25^. A second prefusion-specific epitope, which we call site V, is composed of α2–α3 and β3–β4^23,26^. These secondary structure elements all refold into the HRA that forms the center of the six-helix bundle in postfusion F and therefore these antibodies are likely to exhibit the highest specificity for prefusion F.

Like many other class I fusion proteins, RSV F undergoes proteolytic processing during maturation in the secretory pathway of infected cells. RSV F is synthesized as a single-chain inactive precursor called F_0_ that contains three subunits: F_1_, F_2_, and a 27-amino acid glycopeptide called pep27^27^. This precursor must be cleaved by a furin-like protease to release pep27 and form the mature, fusion-competent protein^27–30^. The C-terminal F_1_ subunit contains the transmembrane domain, two heptad repeats, and an N-terminal fusion peptide. Residues in the F_2_ subunit contribute to fusogenicity of the F protein and possibly the species specificity of RSV^31–35^. In the mature protein, the F_1_ and F_2_ subunits are covalently associated via two disulfide bonds^10,11,30^. Three F_1_–F_2_ protomers then associate via weak intermolecular interactions to form the trimeric, prefusion protein on the surface of the virion. However, these interactions are not sufficient to drive trimerization of the prefusion RSV F ectodomain and therefore engineering of soluble trimeric prefusion F proteins has required the inclusion of artificial trimerization motifs^9,36^.

For class I fusion proteins that lack a fusion-suppressive capping domain, such as the F proteins from pneumoviruses and the related paramyxoviruses^9,37,38^, the extent to which they remain as closed trimers in the context of biological membranes remains unknown. Here, we investigate the dynamic nature of RSV F trimers in solution and on membranes due to the serendipitous discovery of a monoclonal antibody with unique properties. We find that antibody CR9501 favors the opening of soluble, prefusion F trimers and exhibits distinct competition profiles on trimeric and monomeric prefusion F proteins. Analysis of multiple prefusion F structures identifies two conformational states that are related by a breathing motion. We utilize the unique properties of CR9501 to demonstrate that the prefusion RSV F trimer is in a monomer–trimer equilibrium when expressed on the surface of mammalian cells. These findings suggest that prefusion RSV F trimers are dynamic molecules capable of transiently opening and dissociating, a discovery that has implications for the structure and function of class I fusion proteins as well as RSV vaccine development.

## Results

### CR9501 potently neutralizes RSV and binds antigenic site V

**Fig. 1:**
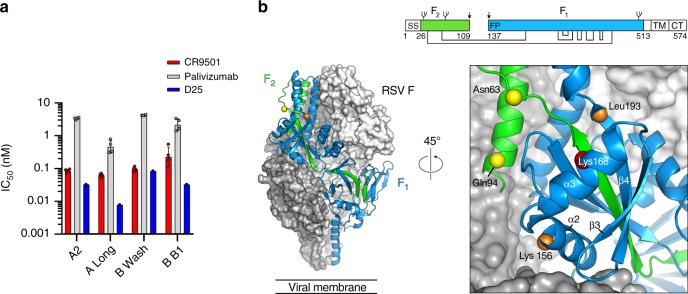
CR9501 potently neutralizes RSV and binds near antigenic site V. **a** Geometric mean neutralization IC_50_ values for CR9501 (red), palivizumab (gray), and D25 (blue). Error bars denote standard deviation of geometric means (*n* = 4 biologically independent experiments with nine technical replicates for CR9501 and palivizumab and *n* = 1 biologically independent experiment with 27–43 technical replicates for D25). **b** Two protomers of prefusion F are shown as molecular surfaces colored gray and white. The third protomer is shown as ribbons, with the F_1_ subunit colored blue and the F_2_ subunit colored green. The Cα atoms of residues identified by shotgun mutagenesis are shown as spheres and colored according to Supplementary Table 1. The inset shows a zoomed view of a 45° rotation about the trimeric axis. A linear depiction of F is shown at the top right, with the F_1_ and F_2_ subunits colored blue and green, respectively. Regions colored white are absent in the soluble protein (SS signal sequence, TM transmembrane, CT cytoplasmic tail). Glycans are shown as branches, disulfide bonds are shown as black lines, and furin cleavage sites are indicated by black arrows

Antibody CR9501 was isolated from the peripheral blood mononuclear cells (PBMCs) of a human donor and found to potently neutralize RSV (Fig. 1a). CR9501 neutralized a small panel of subtype A and B viruses with half-maximal inhibitory concentration (IC_50_) values ranging from 0.06 to 0.23 nM, approximately 7–40-fold more potently than palivizumab. The IC_50_ of CR9501 was similar to that of the prefusion F-specific antibody D25 for three of the four viruses tested. Initial characterization by shotgun mutagenesis identified an epitope that includes site V and portions of the F_2_ subunit (Fig. 1b and Supplementary Table 1), which are in close proximity in the prefusion F structure but are separated by more than 90 Å in postfusion F, suggesting that CR9501 may be prefusion F-specific. Consistent with this result, CR9501 showed undetectable levels of binding to postfusion RSV F as measured by surface plasmon resonance (SPR) (Supplementary Fig. 1).

To better define the epitope, we attempted to crystallize CR9501 in complex with a prefusion-stabilized F ectodomain that contained a C-terminal Foldon trimerization motif (a construct referred to as PR-DM^33^). Because crystallization efforts of this binary complex yielded poorly diffracting crystals, we attempted to generate a ternary complex of prefusion F with CR9501 and motavizumab, an antibody against antigenic site II. Simultaneous addition of CR9501 and motavizumab Fabs resulted in the formation of two complexes with distinct elution volumes on size-exclusion chromatography (Supplementary Fig. 2, black trace). The earlier-eluting peak contained the ternary complex, whereas the later-eluting peak contained RSV F bound only to one Fab. This suggested that there was an order-dependent competition between the antibodies, where initial binding of one antibody prevented binding of the other. To test this hypothesis, we incubated prefusion F with CR9501 prior to the addition of motavizumab. This resulted in a single peak on size-exclusion chromatography with an elution volume corresponding to that of the ternary complex (Supplementary Fig. 2, red trace). In contrast, preincubating prefusion F with motavizumab prior to addition of CR9501 resulted in a decrease of the ternary complex fraction and a corresponding increase in binary complex (Supplementary Fig. 2, blue trace). This order-dependent binding could be due to an antibody-induced or antibody-selected conformational change of RSV F.

### CR9501 and motavizumab exhibit interprotomeric competition

**Fig. 2:**
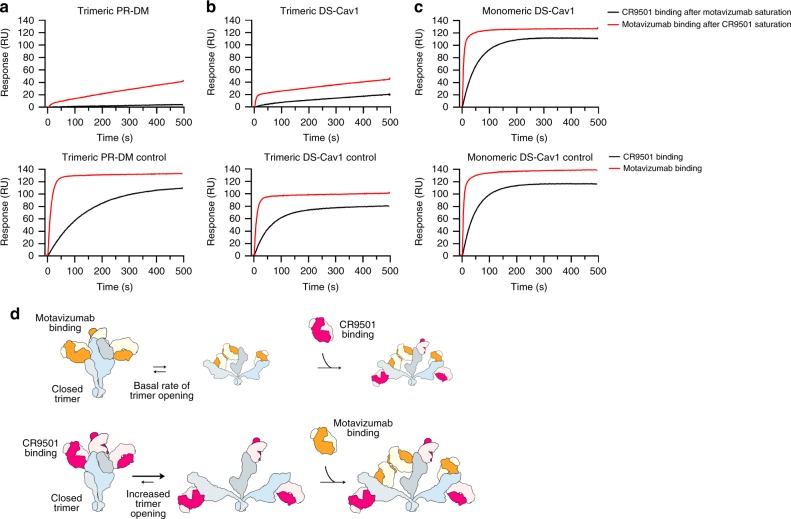
CR9501 enhances opening of prefusion F. Two prefusion F variants that include previously described stabilizing mutations and a trimerization motif—**a** PR-DM or **b** DS-Cav1—or **c** a prefusion-stabilized variant lacking the trimerization motif (monomeric DS-Cav1) were immobilized and bound with saturating levels of either motavizumab or CR9501. A second Fab, either CR9501 (black) or motavizumab (red), was then injected over the surface while measuring the binding response. The bottom row shows the controls in which buffer replaced the first Fab incubation. **d** Model for binding of CR9501 and motavizumab to soluble prefusion F protein illustrating the effects of antibody order-of-addition on trimer opening. The cartoon depicts prefusion F protomers in blue, light gray, and dark gray, CR9501 in pink and light pink, and motavizumab in orange and tan

We had previously predicted that interprotomeric competition may occur between antibodies that recognize antigenic sites II and V^23^. We therefore tested the competition of CR9501 and motavizumab on trimeric and monomeric prefusion F proteins using SPR (Fig. 2). Two previously described variants of trimeric prefusion F (PR-DM and DS-Cav1) were included in these studies. These variants each contain a Foldon trimerization motif but have different stabilizing substitutions. The DS-Cav1 variant contains an additional disulfide bond (S155C/S290C) as well as two cavity-filling mutations (S190F/V207L), whereas PR-DM contains one proline substitution (S215P) and one mutation in the F_2_ subunit (N67I) and is the more stable of the two variants^33,39^. The monomeric prefusion F protein contains the DS-Cav1 stabilizing substitutions but lacks the trimerization domain and elutes at a volume consistent with a single prefusion F protomer on size-exclusion chromatography (Supplementary Fig. 3). For these experiments, the RSV F variants were immobilized on the chip and a sequential injection of CR9501 Fab followed by motavizumab Fab, or motavizumab Fab followed by CR9501 Fab, was performed. For trimeric, prefusion-stabilized variants, saturating the F protein with CR9501 Fab allowed binding of motavizumab Fab (Fig. 2a, b, top panel, red trace), although the response was lower than observed for binding to unliganded prefusion F (Fig. 2a, b, bottom panel, red trace). When trimeric prefusion F proteins were first saturated with motavizumab, little-to-no additional binding was observed upon injection of CR9501 Fab (Fig. 2a, b, top panel, black trace). In contrast, CR9501 and motavizumab did not compete for binding to monomeric prefusion F, as expected (Fig. 2c, top and bottom panels). To verify that this competition profile with CR9501 was unique to antibodies that recognize site II, competition between CR9501 and the site Ø-specific antibody D25 was also measured. Antibodies against site Ø are expected to partially overlap with site V within a single protomer and therefore competition between antibodies recognizing sites Ø and V would not be expected to depend on trimerization of prefusion F. Consistent with this result, D25 competed with CR9501 on both trimeric and monomeric prefusion F (Supplementary Fig. 4). Collectively, these results indicate that CR9501 and motavizumab competition is interprotomeric and suggest that CR9501 binding reduces their competition by favoring opening of the prefusion F trimer.

Based on the results of the competition analysis, we proposed the following model (illustrated in Fig. 2d): (1) the prefusion F trimer exists in an equilibrium between an open and closed state, but the Foldon trimerization motif tethers the protomers and prevents complete dissociation, (2) CR9501 binding favors trimer opening, (3) opening of the CR9501-bound trimer allows motavizumab to bind by relieving the steric hindrance across neighboring protomers, (4) once the ternary complex has formed, the clash between motavizumab and CR9501 prevents the collapse of the complex back to the closed trimeric state, (5) because both Fabs have essentially no off-rate, the equilibrium is driven to a final state comprising each protomer bound to both Fabs.

Closer inspection of the binding rates for the two prefusion F proteins reveals a number of differences that provide insights about the propensity of these two variants to sample the open conformation. First, the rate of CR9501 association with motavizumab-saturated PR-DM, which should reflect the natural rate of PR-DM opening, is very low (Fig. 2a, top panel, black curve). Second, the rate of CR9501 association with motavizumab-saturated DS-Cav1 is higher than observed for PR-DM (Fig. 2b, top panel, black curve), suggesting that DS-Cav1 naturally samples an open state more frequently than does PR-DM. Third, the rate of motavizumab association with CR9501-saturated PR-DM correlates with the CR9501-induced rate of trimer opening because the basal rate of PR-DM opening in the absence of antibodies is negligible (Fig. 2a, top panel, red curve). Finally, two different association rates are visible in the association of motavizumab with CR9501-saturated DS-Cav1 (Fig. 2b, top panel, red curve). The first rate likely represents the fraction of DS-Cav1 that is already present in an open conformation and can readily bind to motavizumab upon injection. This rate is very fast, similar to the rate of motavizumab binding to unliganded DS-Cav1 (Fig. 2b, bottom panel, red curve). The second, slower rate correlates with the rate of DS-Cav1 opening and is only slightly increased in the presence of CR9501, again suggesting that DS-Cav1 samples an open conformation more often than PR-DM. Consistent with this hypothesis, DS-Cav1 incubated with motavizumab prior to addition of CR9501 resulted in almost entirely ternary complex as measured by SEC (Supplementary Fig. 2, blue trace).

### Structure reveals interprotomeric clash between antibodies

**Fig. 3:**
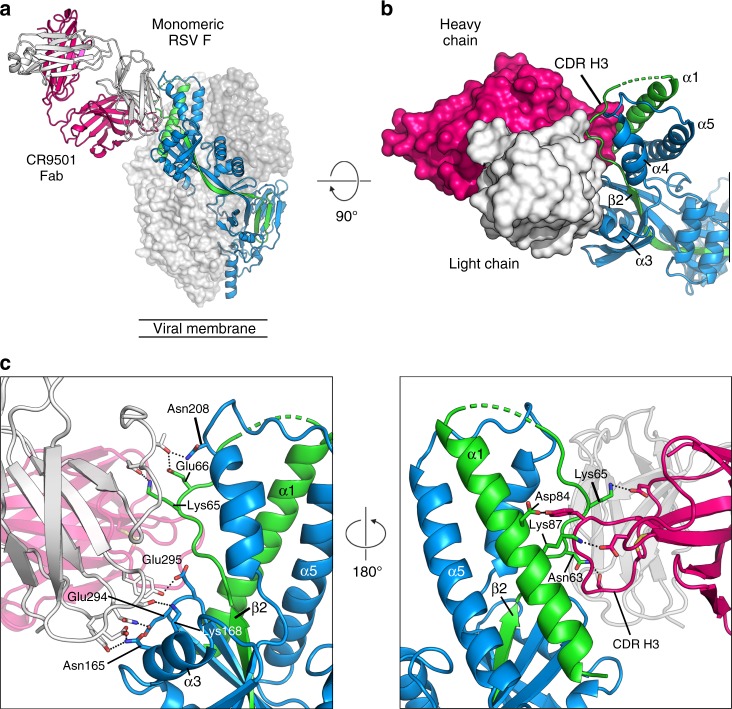
Crystal structure of CR9501 bound to monomeric prefusion F. **a** Monomeric prefusion F is shown as ribbons, with the F_1_ subunit colored blue and the F_2_ subunit colored green. The CR9501 heavy and light chains are shown as pink and white ribbons, respectively. The two additional protomers of the prefusion F trimer are generated using the C3 symmetry observed in previous crystal structures and shown as transparent molecular surfaces. **b** A 90° rotation about the horizontal axis with the CR9501 variable domain shown as molecular surfaces illustrates the insertion of the CDR H3 between two regions of the F_2_ subunit. **c** A zoomed-in view of the orientation in (**a**) (left) and a 180° rotation about the trimeric axis (right). Side chains involved in hydrogen bonds or salt bridges are shown as sticks with oxygen and nitrogen atoms colored red and blue, respectively. Hydrogen bonds and salt bridges are depicted as black dotted lines

To avoid conformational heterogeneity resulting from CR9501-induced opening of prefusion F trimers, we sought to determine a CR9501-bound F structure by using the monomeric variant of prefusion F. This complex formed crystals in space group *P*2_1_2_1_2_1_ that diffracted X-rays to 3.3 Å resolution (Supplementary Table 2). The structure revealed that CR9501 recognizes antigenic site V, but also makes substantial contacts with the F_2_ subunit (Fig. 3a), consistent with the results of shotgun mutagenesis. The third heavy chain complementarity-determining region (CDR H3) of CR9501 is inserted between two elements of the F_2_ subunit—the α1 helix and the loop that connects this helix to β2 (Fig. 3b). CR9501 uses both the heavy and light chain CDRs to contact five discrete regions of the linear RSV F protein sequence that are in close proximity in the prefusion F conformation (Fig. 3c). The light chain interacts with four of these regions, forming a salt bridge with Lys65 and a hydrogen bond with Glu66, both of which are on the loop connecting β2 and α1 in the F_2_ subunit. In addition, the light chain forms hydrogen bonds with two residues in the α3 helix (Asn165 and Lys168) and on the loop connecting β5 and β6 (Glu294 and Glu295). The heavy chain makes fewer contacts, the majority of which are with the F_2_ subunit. The disulfide-stabilized CDR H3 forms hydrogen bonds with Asn63 and Asp84 and a salt bridge with Lys65. A salt bridge is also formed between CDR H1 and Lys87 of the α1 helix. In the postfusion F conformation, α3 would be separated from the other elements of the CR9501 epitope by more than 90 Å, suggesting that CR9501 neutralizes RSV by trapping the prefusion conformation of F—acting as a staple that links the α3 helix to more conformationally stable regions of the prefusion F protein.

**Fig. 4:**
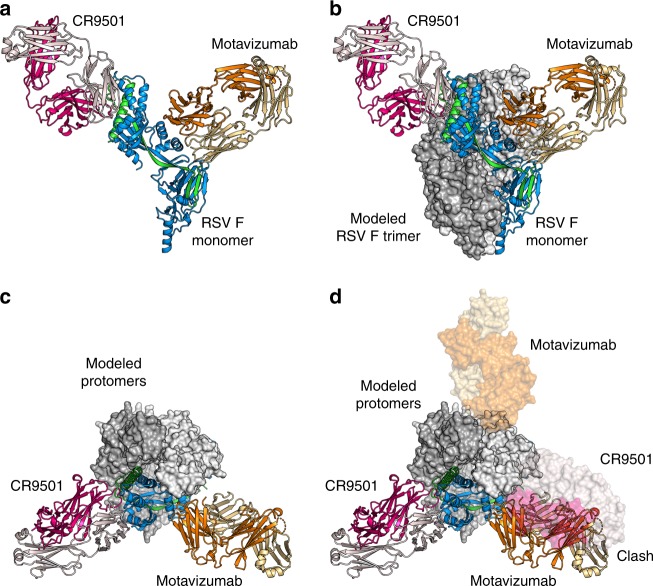
CR9501 and motavizumab Fabs cannot simultaneously bind to closed, trimeric F. **a** Monomeric prefusion F-CR9501 structure is shown in ribbons with the F_1_ subunit colored blue and the F_2_ subunit colored green. The CR9501 heavy and light chains are shown as dark pink and light pink ribbons, respectively. Motavizumab Fab is modeled onto the prefusion F monomer using the previously solved structure (PDB ID:3IXT) and is shown in ribbons with the heavy and light chains colored orange and tan, respectively. **b** The two additional protomers of the prefusion F trimer were generated using the C3 symmetry observed in previous crystal structures and are shown as molecular surfaces. **c** A 90° rotation about the horizontal axis of the complex shown in (**b**), viewed looking toward the viral membrane. **d** A second asymmetric unit from the crystal structure, composed of one prefusion F protomer and one CR9501 Fab, and one modeled motavizumab Fab were aligned with a neighboring protomer of the biological trimer and are shown as transparent molecular surfaces. An interprotomeric clash is observed between CR9501 and motavizumab in the context of the prefusion F trimer

We generated the biological RSV F trimer by applying the threefold symmetry observed in previous structures to the monomeric complex. This model revealed that the CR9501 Fab bound to one RSV F protomer would clash substantially with the motavizumab Fab bound to the neighboring protomer (Fig. 4). This indicates that in order for the ternary complex to form, the prefusion F trimer must be in an open conformation that alleviates the interprotomeric clash between these two antibodies (Supplementary Movie 1), as predicted based on the results of the competition analysis.

### CR9501 causes prefusion F trimers to splay open in solution

**Fig. 5:**
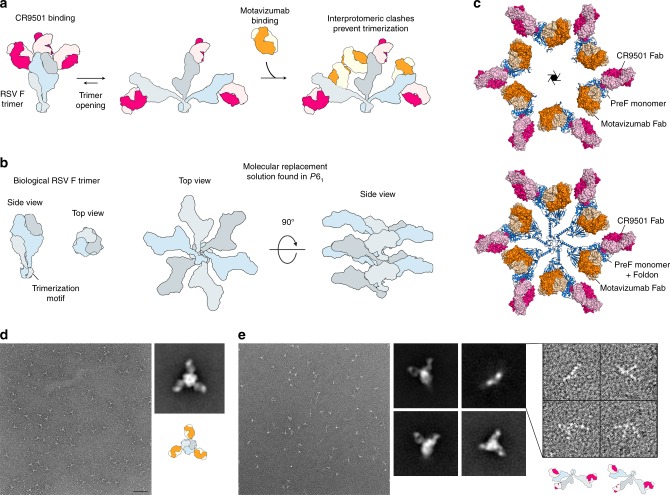
Structural evidence for CR9501-enhanced opening of prefusion F trimers. **a** Model illustrating the strategy used to generate trimeric prefusion F bound simultaneously by CR9501 and motavizumab, showing prefusion F protomers in blue, light gray and dark gray, CR9501 in pink and light pink, and motavizumab in orange and tan. **b** Cartoon depicts the biological RSV F trimer (left) and the molecular replacement solution obtained in space group *P*6_1_ (right), shown without Fabs for clarity. Prefusion F protomers are colored blue, light gray, and dark gray. **c** The molecular replacement solution from the crystal structure of prefusion F bound to CR9501 and motavizumab, with six copies of the asymmetric unit shown and the sixfold screw axis indicated in the center (top). Prefusion F is shown as blue ribbons and motavizumab and CR9501 Fabs are shown as molecular surfaces. CR9501 heavy and light chains are colored dark pink and light pink and the motavizumab Fab heavy and light chains are colored orange and tan, respectively. The Foldon motif was modeled on to the C-terminus of each monomer, using a previously determined prefusion F structure in which this domain is visible (PDB ID: 4MMV) (bottom). **d** Negative-stain EM of the prefusion F-motavizumab complex showed primarily top-down views of the closed, trimeric complex (scale bar = 50 nm). A representative class average shows a closed trimer with three Fabs bound and the cartoon illustrates the orientation viewed in the class average. **e** Negative-stain EM of the prefusion F-CR9501 complex revealed multiple particle classes. Three representative class averages show different orientations of the closed, trimeric complex, whereas the fourth comprised individual monomers within splayed-open trimers. The inset shows a subset of the splayed-open trimers used to generate the fourth class average (right) and the cartoons illustrate the orientation of the splayed-open trimer thought to be present in the particles shown

To validate the model proposed above, we sought to capture the prefusion F trimer in the splayed-open state. We therefore preincubated PR-DM with CR9501 before addition of motavizumab to drive the equilibrium toward the fully open conformation (Fig. 5a). This complex was analyzed by negative-stain EM, but because the orientation of each protomer relative to the other protomers in a splayed-open trimer is variable, 2D classification did not yield a splayed-open trimer but rather a single protomer bound by two Fabs (Supplementary Fig. 5). Therefore, we crystallized the complex, in hopes of capturing a static view of a splayed-open trimer. The complex formed crystals in space group *P*6_1_ that diffracted X-rays to 4.1 Å resolution (Supplementary Table 2). Molecular replacement identified one RSV F protomer bound to one CR9501 Fab and one motavizumab Fab in the asymmetric unit. Because *P*6_1_ lacks a non-screw threefold axis, a biological trimer could not be generated by symmetry operations, indicating that the protomers dissociated during (or prior to) crystallization (Fig. 5b, c). Although the density for the C-terminal portion of RSV F is not visible, the orientation of each monomer in the unit cell is compatible with the distance required for Foldon to remain trimeric while also allowing the dissociated protomers to pack along the sixfold screw axis (Fig. 5c). Thus, our crystallographic analysis again suggests the presence of a splayed-open trimer.

Because crystallization can select for conformations that do not predominate in solution, we sought to estimate the extent to which CR9501 binding alters the monomer–trimer equilibrium using another method. We performed negative-stain EM on CR9501-bound prefusion F (PR-DM) and compared it to the negative-stain EM for motavizumab-bound prefusion F (Fig. 5d, e). Micrographs for the prefusion F-motavizumab complex were primarily composed of top-down views of the trimer bound by three Fabs (Fig. 5d). In contrast, the micrographs of the prefusion F-CR9501 complex contained three distinct particle classes in which the CR9501-bound trimer resembled previously characterized Fab-bound prefusion F trimers and one class composed of CR9501-bound trimers that appeared to be completely splayed open, tethered only by the trimerization motif (Fig. 5e). To verify that the closed conformation of prefusion F observed in the motavizumab-bound samples was not due to trimer-stabilizing effects of this antibody, we coexpressed monomeric prefusion F with motavizumab and analyzed the resulting complex by size-exclusion chromatography (Supplementary Fig. 6). This complex eluted at a volume corresponding to a Fab-bound monomer. In contrast, coexpression with the quaternary-specific antibody AM14 resulted in an elution profile corresponding to a Fab-bound trimer, consistent with published results^40^. Collectively, these results suggest that the trimeric and monomeric forms of soluble prefusion F exist in an equilibrium and demonstrate that binding of CR9501 favors trimer dissociation.

### CR9501-bound F resembles a natural prefusion state

**Fig. 6:**
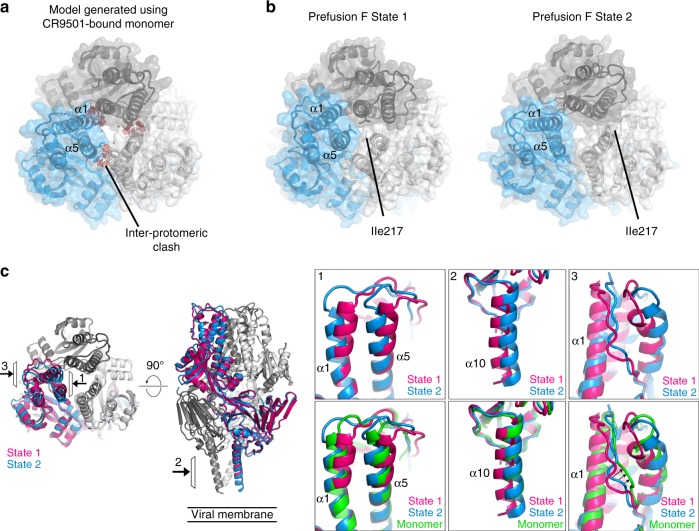
CR9501-bound F resembles a naturally occurring prefusion state. **a** C3 symmetry was applied to the monomeric prefusion F-CR9501 structure to generate a prefusion F trimer. The resulting trimer is shown as ribbons and transparent molecular surfaces, with the three protomers colored blue, gray, and white, viewed looking toward the viral membrane. Predicted clashes between protomers are indicated with red. **b** Two states of prefusion F identified in 26 previously determined structures are shown and colored as in (**a**). **c** The two states of prefusion F are shown in pink and blue ribbons and viewed looking toward (left) or along (right) the viral membrane. The top insets show the views denoted by the arrows at (1) the trimer apex viewed from the central cavity, (2) the heptad repeat B and (3) the F_2_ subunit. The bottom insets are the same but include the monomer from the CR9501-bound structure shown in green

To determine the structural basis for CR9501-enhanced trimer dissociation, we compared the CR9501-bound prefusion F monomer structure to a single protomer of a previously solved trimeric prefusion F structure (PDB ID: 4MMU). These structures were similar, with an RMSD of 1.7 Å for 430 Cα atoms. However, despite the high overall similarity, several differences were observed. A rigid-body motion between the trimer apex and the membrane-proximal region of the protein was present in the monomeric structure. This resulted in a change in the orientation of the α1 and α5 helices at the apex of the protein, and a small change in the orientation of antigenic site IV and the α10 helix at the base of the CR9501-bound monomer compared to the 4MMU structure. When three monomers from the CR9501-bound structure were used to model the prefusion F trimer, a small interprotomeric clash between helices α1 and α5 of adjacent protomers was visible (Fig. 6a). Although it is possible that this clash could contribute to destabilization of the prefusion F trimer, it is not possible to determine if this clash is a result of CR9501 binding, or if this change in the apex of prefusion F simply represents a different conformation that can be adopted by monomeric prefusion F when it is not constrained by the presence of other protomers.

Because the crystal structures of multiple trimeric prefusion RSV F proteins have recently been solved, we compared these structures to determine if the conformational changes we observed are specific to the CR9501-bound structure. Combining the static snapshots from 26 previously solved prefusion F structures revealed trimer flexibility at both the trimer apex and the membrane-proximal region (Supplementary Movies 2 and 3). In each of the structures analyzed, the α1 and α5 helices adopt one of two distinct orientations (Fig. 6b, c, Supplementary Table 3). In the first orientation (State 1), the α1 and α5 helices are closely packed, with the membrane-distal region of the α5 helix from one protomer contacting both the α1 and α5 helices from the neighboring protomer. In this conformation, Ile217 is oriented toward the center of the trimer apex and shields access to the central cavity. In the second arrangement (State 2), the top portion of the α5 and α1 helices are tilted by approximately 20° and 10°, respectively, resulting in a decrease in the interprotomeric contacts between α1 and α5. In this state, Ile217 is oriented away from the central threefold axis and no longer shields the central cavity. Analysis of representative trimers from each state using the PDBePISA server showed that a State 1 trimer buries an average of 2331 Å^2^ in each interprotomeric interface, with a predicted Δ*G* = −18.5 kcal mol^−1^, whereas a State 2 trimer buries 1928 Å^2^, with a predicted Δ*G* = −15.0 kcal mol^−1^. The membrane-proximal region of the prefusion F trimer also displayed a small degree of flexibility, with a continuum of orientations in which the α10 helix shifts by up to 2 Å away from the central threefold axis of the trimer (Fig. 6c). The crystal structures in which prefusion F adopted the State 2 conformation all display some degree of movement of the α10 helix away from the central threefold axis, suggesting that changes at the trimer apex may be coupled to those in the membrane-proximal region. In addition, the position of the CR9501 CDR H3 between two portions of the F_2_ subunit results in a small movement of this loop away from the α1 helix compared with the previously determined structure. Comparison to two other RSV F structures (PDB IDs: 4JHW and 4MMT) indicated that although the F_2_ subunit exists in multiple conformations, the degree of movement away from the α1 helix is largest in CR9501-bound RSV F (Fig. 6c).

Comparing the rigid-body movements identified in the CR9501-bound monomer to those of the two prefusion F states identified above revealed that the conformation of the CR9501-bound monomer most closely resembles that of the State 2 trimer (Fig. 6a–c). The α1 and α5 helices are tilted away from the apex of the neighboring protomer, although to a lesser degree than what was observed for trimeric proteins, resulting in loss of inter-protomeric contacts between α1 and α5 and orientation of Ile217 outward to expose the central cavity. Consistent with the subtle differences between the CR9501-bound monomer and the trimeric prefusion F structures, CR9501 displayed high-affinity binding for both trimeric and monomeric prefusion F (Supplementary Fig. 7). Together these analyses suggest that the rigid-body motion observed in the CR9501-bound monomer represents a naturally occurring conformation of prefusion F rather than a CR9501-induced change, and that there are at least two conformations of the prefusion F trimer, one of which has reduced interprotomeric stability.

### RSV F trimers open and dissociate at the cell surface

**Fig. 7:**
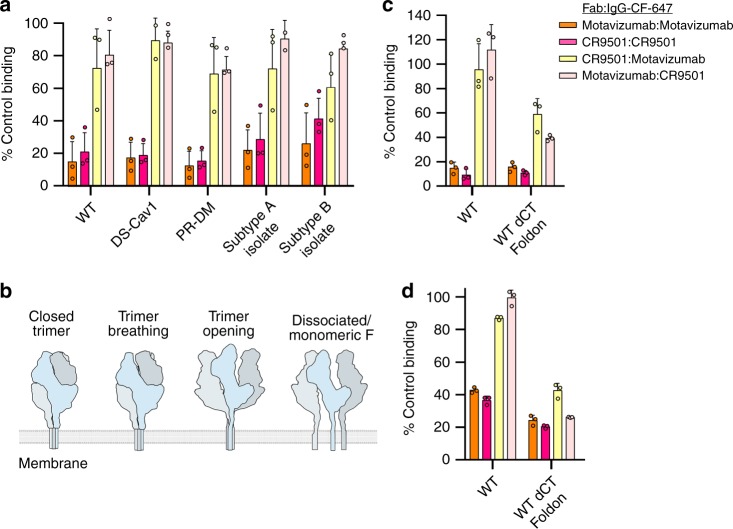
RSV F trimers open and dissociate at the cell surface. **a** Full-length RSV F proteins were expressed on the surface of FreeStyle 293-F cells and incubated with either CR9501 Fab or motavizumab Fab. Cells were then stained with CF-647-labeled CR9501 IgG or CF-647-labeled motavizumab IgG and binding was analyzed by flow cytometry. **b** A cartoon depicts prefusion F protomers in blue, light gray, and gray and shows four possible states of the prefusion F trimer, two of which could allow simultaneous binding of motavizumab and CR9501. **c** Full-length RSV F or a variant in which the cytoplasmic tail was replaced with a Foldon trimerization motif was expressed on the surface of FreeStyle 293-F cells and stained as in (**a**). **d** Same as (**c**), but with RSV F and the Foldon variant expressed on the surface of HeLa cells. Bar graphs show the mean and error bars indicate the standard deviation (*n* = 3 biologically independent experiments with two technical replicates)

The unique competition profiles observed for CR9501 and motavizumab on trimeric and monomeric variants of prefusion F suggested that CR9501 could be used as a reagent to probe trimer opening of full-length RSV F on the surface of cells. FreeStyle 293-F cells were transfected with a vector encoding full-length wild-type F and incubated with competitor Fab or buffer before being incubated with an CF-647-labeled IgG. CR9501 Fab preincubation reduced the signal observed in cells stained with CF-647-labeled CR9501 IgG by nearly 80% compared with buffer-only preincubation (Fig. 7a, dark pink). A similar result was observed for motavizumab Fab preincubation before staining with CF-647-labeled motavizumab IgG (Fig. 7a, orange). Preincubation of the cells with CR9501 Fab blocked only ~30% of CF-647-labeled motavizumab IgG binding (Fig. 7a, yellow), consistent with the model above in which CR9501 binding favors opening of the trimer and relieves the interprotomeric clash with motavizumab. However, in contrast to what was observed with soluble trimeric prefusion F proteins, motavizumab preincubation blocked binding of CF-647-labeled CR9501 IgG by less than 20% (Fig. 7a, light pink). These results indicate that the full-length F protein on the surface of cells samples an open conformation compatible with the binding of both CR9501 and motavizumab.

To verify that the stabilizing mutations present in the soluble proteins were not responsible for order-of-addition dependence observed previously, these same stabilizing mutations were incorporated into full-length F and the resulting variants were expressed on the surface of cells. Similar to the wild-type protein, very little competition was observed between CR9501 and motavizumab for either order of antibody addition (Fig. 7a). To determine if this phenomenon was restricted to lab-adapted RSV strains, we tested two additional variants of the full-length proteins that were generated using the F protein sequences from two clinical isolates of RSV. CR9501 and motavizumab again showed low levels of competition for binding to cells expressing these variants (Fig. 7a).

Our results thus far indicated that full-length RSV F samples a conformation or oligomeric state on the surface of cells compatible with the binding of both antibodies. However, our data could not yet distinguish between a model in which RSV F is partially open or completely dissociated (monomeric), which largely depends upon whether the transmembrane domains are associated (Fig. 7b). Thus, we performed an additional experiment in which the cytoplasmic tail of F was replaced with a Foldon trimerization motif, and CR9501 and motavizumab competition was again measured on the surface of HEK293-F cells (Fig. 7c). We reasoned that if prefusion F trimers are partially open on the surface of cells, inclusion of this domain should not impact the competition of CR9501 and motavizumab, since it would be redundant with the presence of the associated transmembrane domains. In contrast, if the trimers dissociated at the cell surface, inclusion of the Foldon domain should increase competition between CR9501 and motavizumab, since Foldon would favor trimerization of the protomers. When F was fused to the Foldon motif, increased competition between CR9501 and motavizumab was observed (Fig. 7c, yellow and pink), indicating that a fraction of prefusion F is monomeric at the cell surface.

Although competition between CR9501 and motavizumab was higher in the Foldon-fused variant than in wild-type RSV F, binding was not reduced to the level observed for the control samples (Fig. 7c, orange and dark pink), suggesting that partial trimer opening also occurs. To verify that this property was not restricted to suspension cell lines, we repeated the competition assay with RSV F and the Foldon-fused variant expressed on the surface of adherent HeLa cells and obtained similar results (Fig. 7d). To rule out the possibility that RSV F is entirely monomeric when expressed at the cell surface, we measured binding to RSV F-transfected cells of the quaternary-specific antibody AM14^40^ and a prefusion-specific antibody (AM22^9^) that can bind to both monomeric and trimeric F (Supplementary Fig. 8). We reasoned that if there were a static pool of monomeric RSV F at the cell surface, the AM14 signal would be much lower than that of AM22. However, AM14 binding was similar to AM22, indicating that full-length F is in a monomer−trimer equilibrium at the cell surface.

## Discussion

The biochemical assays and structural analysis presented here demonstrate that CR9501 represents a new class of RSV-neutralizing antibody that enhances dissociation of trimeric prefusion F while also preventing the conformational changes required for conversion of prefusion F into the postfusion conformation. We have recently determined that a large proportion of the neutralizing antibody response in healthy adults target this prefusion-specific antigenic site^23^, but only one other site V-reactive antibody—hRSV90—has been structurally characterized thus far^26^. Although both CR9501 and hRSV90 contact antigenic site V, CR9501 is rotated by ~60° compared with hRSV90 (Supplementary Fig. 9), resulting in substantial contacts with the F_2_ subunit. Similar effects on trimerization were not reported for hRSV90, indicating that the additional contacts made between CR9501 and the F_2_ subunit could be responsible for the trimer disassembly observed here. Previous studies have implicated the F_2_ subunit in species specificity of RSV and in fusogenicity of F^31,32^. However, the conformational changes observed in the CR9501-bound monomer are subtle, in contrast to what has been observed in monomeric structures of antibody-bound influenza hemagglutinin (HA) in which conformational changes indicative of triggering are present^41–44^. This is likely because CR9501 also contacts the α2–α3 helices, which would prevent conformational changes associated with fusion. It therefore remains possible that interactions with the F_2_ subunit are linked to monomerization, triggering, or both, but these changes could be countered in the CR9501-bound structure by the stabilizing contacts made with other regions of the protein.

Our analysis of the many previously determined RSV F structures suggests that the trimeric, prefusion F protein samples at least two conformations, one of which is less compact and could favor trimer opening. The small changes that occur between the two states of the prefusion F trimer are reminiscent of the breathing motions that have been described for the envelope (Env) protein of HIV-1, which have been observed in soluble proteins and on the surface of virions^45,46^. This conformational flexibility is required for binding of the HIV-1 receptor and coreceptor, which prime Env for fusion^45–47^. Mutations that stabilize the trimeric interface at the base of Env can limit breathing at the trimer apex and prevent the conformational changes required for receptor binding^48^. Antibodies that cause Env trimer dissociation have been described and it was proposed that binding of these antibodies may depend on the same breathing motions required for receptor binding^49^. Similar to CR9501, these antibodies neutralize HIV-1 and recognize both monomeric and trimeric Env proteins, suggesting that susceptibility to antibody-induced changes in oligomeric state may be a common feature among class I fusion proteins, particularly those that exhibit conformational flexibility.

Prefusion ectodomains of RSV F, as well as a number of other class I fusion proteins, are monomeric in the absence of a trimerization domain, suggesting that interprotomeric interactions are weak in these proteins^9,37,38^. It has therefore been assumed that the restriction of the protomers within the 2D membrane increases the local concentration of these proteins sufficiently to favor trimerization. Our results suggest that for RSV F this may not always be true, and that prefusion F exists in a monomer–trimer equilibrium, even when embedded in a lipid bilayer. We note that cryo-electron tomography studies of budding RSV particles have only identified density consistent with trimeric prefusion F at the surface of filamentous viral particles^50,51^. This may be because monomeric proteins are present at the viral surface but are too small to be visualized by tomography, or that the RSV matrix protein layer, present ~5 nm below the viral membrane, plays a role in the trimerization of RSV F. This would be consistent with previous studies suggesting that the matrix protein and the F cytoplasmic tail interact^52^. Interestingly, a monomer-specific antibody isolated against HA binds to influenza-infected cells^53^, indicating that HA may also sample an open conformation at the cell membrane. This finding suggests that trimer opening at the cell surface may not be unique to RSV and could represent a previously unappreciated property of certain class I fusion proteins. These results also have implications for RSV vaccine design because several leading candidates are based on trimeric prefusion-stabilized F antigens. Studies will be needed to assess the relationship between transient trimer opening and immunogenicity.

## Methods

### Identification of anti-RSV F antibodies

Neutralizing monoclonal antibodies against RSV F protein were recovered from mature B cells isolated from PBMCs obtained through the San Diego Blood Bank. Samples obtained from human volunteers followed protocols approved by the San Diego Blood Bank Review Board and informed consent was obtained from the donors prior to the blood donation. In short, CD22^+^ B cells were isolated by immunomagnetic positive selection (Miltenyi) from donor PBMCs and transformed with Epstein-Barr virus, followed by stimulation for 5 days with CpG and recombinant human interleukin 2 (IL-2). Two weeks later, supernatants were tested by enzyme-linked immunosorbent assay (ELISA) against RSV F-transfected cells. Variable-heavy (VH) and variable-light (VL) chain genes were cloned from oligoclonal B-cell cultures corresponding to antigen-reactive wells using pooled primers described in patent application US20130177573A1, and specificity of the cloned antibodies was confirmed by ELISA.

### Expression of antibodies utilized in neutralization assays

The sequences obtained from each VH and VL of the antibody clones were codon-optimized for expression in human cells at GeneArt/Invitrogen. The variable regions of these functional variants were subsequently cloned directly by restriction digest for expression in the IgG expression vectors pCP9-kappa and pCP9-gamma. Vectors encoding the IgGs were transfected into PER.C6® cells and the antibodies were purified from cell supernatants using POROS Mabcapture A chromatography (Applied Biosystems). The antibodies were then exchanged into 50 mM sodium acetate, 50 mM sodium chloride, pH 5.5. The quality of each antibody was confirmed by size-exclusion chromatography, SDS-PAGE and isoelectric focusing.

### Neutralization assay

RSV-susceptible VERO cells (ATCC-CCL-81) were seeded at a density of 3 × 10^4^ cells well^−1^ in a 96-well format. The following day 1200 plaque-forming units of virus were incubated with serial dilutions of the RSV antibodies for 1 h at 37 °C before addition to the cells and incubation for 24 h. The media was then refreshed, and the cells were incubated for 48 h. The cells were fixed with 80% acetone and virus growth was detected using a biotinylated anti-F protein antibody coupled with HRP-conjugated streptavidin. The wells were washed again, and turnover of the 3,3′,5,5′-tetramethylbenzidine substrate was measured at an optical density of 450 nm (OD_450_). VNA titers were calculated as the antibody concentration that caused 50% reduction in the OD_450_, expressed as IC_50_. Four biologically independent experiments with nine technical replicates were performed for CR9501 and palivizumab and one independent experiment with 27–43 technical replicates was performed for D25.

### RSV F mutation library and immunofluorescence assay

Epitope mapping by mutagenesis was performed by Integral Molecular. A shotgun mutagenesis library of RSV F was created using a previously published method^54^. The parental plasmid expressing RSV F A2 strain (GenBank: FJ614814.1) polyprotein was used as a template to make a library of random mutations across RSV F, created using PCR-based mutagenesis (Diversify PCR Random Mutagenesis Kit, Clontech) plus 39 additional mutants. Each mutant clone was sequence verified. A complete mutation library was assembled by selection of at least two mutant clones per residue, preferably representing a conserved and nonconserved residue at each position.

Mutation libraries and controls were expressed in human HEK-293T cells (ATCC-CRL-3216). Twenty-two hours posttransfection cells were washed and fixed in 4% (wt/vol) paraformaldehyde, incubated with 0.5 µgmL^−1^ of CR9501 or CR9502 IgG^33^, followed by AlexaFluor 488-conjugated secondary antibody (Jackson ImmunoResearch). Microplates were measured using the Intellicyt HTFC screening system. Antibody reactivities against each mutant F protein clone were calculated relative to wild-type protein reactivity by subtracting the signal from mock-transfected controls and normalizing to the signal from wild-type F-transfected controls. Binding of CR9501 was also compared with a polyclonal serum, palivizumab, and CR9502, representing different epitopes, to confirm that the expressed mutant F proteins were not misfolded.

### Antibody production for crystallization, SPR, and cytometry

Plasmids encoding the heavy and light chain of CR9501 or motavizumab antibodies were cotransfected into FreeStyle-293F cells (ThermoFisher Scientific, Cat. No. R79007). Cell supernatants were harvested after 6 days and antibodies were purified using protein A resin, followed by size-exclusion chromatography using a Superdex 200 column (GE Healthcare) in buffer containing 2 mM Tris pH 8.0, 200 mM NaCl and 0.02% NaN_3_ or phosphate-buffered saline (PBS) with 0.02% NaN_3_.

To generate motavizumab and CR9501 Fabs, a 1 mg mL^−1^ solution of each antibody was digested with 1:4000 (wt/wt) of Lys-C overnight at 37 °C. Undigested antibody and digested Fc were separated from Fabs using protein A resin, and the flow-through was further purified by size-exclusion chromatography using a Superdex 75 column (GE Healthcare) in buffer containing 2 mM Tris pH 8.0, 200 mM NaCl and 0.02% NaN_3_ or phosphate-buffered saline with 0.02% NaN_3_.

### Production of protein complexes for crystallization and SPR

Trimeric prefusion F (PR-DM)^33^ comprised residues 1–513 from RSV F strain A2 with N67I/S215P mutations and a C-terminal fibritin trimerization motif (Foldon), HRV 3C cleavage site, 8× His-tag and StrepTagII. A second variant of trimeric prefusion F called DS-Cav1^39^ is composed of residues 1–513 from RSV F strain A2 with S155C/S290C/S190F/V207L substitutions and a C-terminal Foldon trimerization motif, thrombin cleavage site, 8× His-tag and StrepTagII. Monomeric prefusion F is identical to DS-Cav1 but lacks the Foldon trimerization motif. Vectors encoding each were transfected into FreeStyle 293-F cells (ThermoFisher Scientific, Cat. No. R79007). For proteins utilized in crystallization, kifunensine was added to the cell suspension approximately 4 h after transfection to a final concentration of 5 μM. Proteins were purified from cell supernatants using StrepTactin resin (IBA), then digested with 5–10% (wt/wt) EndoH to remove *N*-linked glycans, followed by cleavage with 10 units mg^−1^ of HRV 3C to remove tags. The protein was further purified by size-exclusion chromatography using a Superdex 200 column (GE Healthcare) in buffer containing 2 mM Tris pH 8.0, 200 mM NaCl and 0.02% NaN_3_.

Ternary prefusion F-motavizumab-CR9501 complex was generated by first incubating PR-DM with a 1.5-fold molar excess of CR9501 Fab at room temperature for 1 h, followed by the addition of 1.5-fold molar excess of motavizumab Fab and incubation at room temperature for 30 min. The complexes were then separated from excess Fabs using a Superdex 200 Increase column (GE Healthcare). The monomeric prefusion F-CR9501 complex was produced by incubating monomeric DS-Cav1 with a 1.5-fold molar excess of CR9501 Fab before separating the complex from excess Fabs using a Superdex 200 Increase column (GE Healthcare).

### Analytical size-exclusion chromatography

PR-DM or DS-Cav1 were incubated with a 1.5-fold molar excess of CR9501 Fab at room temperature for 1 h, followed by the addition of 1.5-fold molar excess of motavizumab Fab and incubation at room temperature for 30 min. Complexes were also generated by first incubating PR-DM or DS-Cav1 with motavizumab Fab for 1 h before incubation with CR9501 for 30 min. For simultaneous addition, both Fabs were incubated with PR-DM or DS-Cav1 for 30 min. The complexes were then analyzed using a Superdex 200 Increase column (GE Healthcare) in buffer containing 2 mM Tris pH 8.0, 200 mM NaCl and 0.02% NaN_3_.

Monomeric prefusion F (DS-Cav1) was coexpressed with either motavizumab Fab or AM14 Fab and furin in FreeStyle 293-F cells (ThermoFisher Scientific, Cat. No. R79007) and purified from cell supernatants using Ni-NTA resin (ThermoScientific). The complexes were then analyzed using a Superdex 200 Increase column (GE Healthcare) in buffer containing 2 mM Tris pH 8.0, 200 mM NaCl and 0.02% NaN_3_.

### Crystallization and data collection

The monomeric prefusion F-CR9501 complex was crystallized by sitting-drop vapor diffusion by mixing 100 nL of protein (4.2 mg mL^−1^) with 50 nL of a reservoir solution composed of 14.3% (w/v) polyethylene glycol (PEG) 8000, 2.4% (v/v) 2-methyl-2,4-pentanediol (MPD), 0.1 M sodium citrate pH 5.5 and 0.1 M sodium malonate pH 7.0. Crystals were soaked in reservoir solution supplemented with 30% (v/v) ethylene glycol and plunge frozen in liquid nitrogen. Data were collected to 3.3 Å at the SBC beamline 19-ID using a wavelength of 0.9792 Å (Advanced Photon Source, Argonne National Laboratory).

Crystals of the ternary prefusion F-motavizumab-CR9501 complex were produced by hanging-drop vapor diffusion by mixing 0.67 µL of protein (4.69 mg mL^−1^) with 1.33 µL of a reservoir solution containing 30% (v/v) PEG 400, 0.19 M ammonium sulfate, 3.1% (w/v) PEG 8000 and 0.1 M Tris pH 8.5. Crystals were transferred into a solution containing reservoir supplemented with 20% (v/v) glycerol and suspended over an identical reservoir solution for approximately 9 h before plunge freezing in liquid nitrogen. Diffraction data were collected to 4.1 Å at the SBC beamline 19-ID using a wavelength of 0.9793 Å (Advanced Photon Source, Argonne National Laboratory).

### Structure determination, model building, and refinement

Software used for processing of X-ray diffraction data was curated by SBGrid and accessed through the CCP4i interface^55–57^. Data were indexed and integrated using iMOSFLM^58^ before merging and scaling with AIMLESS^59^. PHASER^60^ was used to identify molecular replacement solutions, and the structures were refined using PHENIX^61^ and built manually using Coot^62^. Data collection and refinement statistics for the two crystal structures are shown in Supplementary Table 2 and a stereo image of a portion of the electron density map is shown in Supplementary Fig. 10.

The monomeric prefusion F-CR9501 complex crystallized in space group *P*2_1_2_1_2_1_ and a molecular replacement solution was found using a prefusion F protomer (PDB ID: 5EA4), and the CR9501 Fab from the ternary complex as search models. The asymmetric unit contained two prefusion F protomers and two CR9501 Fabs. The model was built manually in Coot and refined in PHENIX using NCS torsion restraints to an *R*_work_/*R*_free_ of 21.2/25.9%.

The ternary prefusion F-motavizumab-CR9501 complex formed crystals in space group *P*6_1_, and a molecular replacement solution was found using one prefusion F protomer (PDB ID: 5C69), one motavizumab Fab (PDB ID: 3QWO), the light chain from D25 (PDB ID: 4JHA), and the heavy chain from N60-I3 Fab (PDB ID: 4RFO) as search models. The asymmetric unit contained one prefusion F protomer, one motavizumab Fab, and one CR9501 Fab. Electron density was not present for the membrane-proximal region of prefusion F and therefore this portion of the structure is absent in our model. The remainder of the model was built manually in Coot and refined in PHENIX using reference-model restraints to an *R*_work_/*R*_free_ of 26.2/30.6%.

### Negative-stain electron microscopy

Carbon-coated grids (EMS) were glow-discharged before 4.8 µL of PR-DM-CR9501 complex or PR-DM-motavizumab complex (0.02 mg mL^−1^) was applied to the grid and allowed to incubate for 30 s. The grids were rinsed with a buffer containing 2 mM Tris pH 8.0, 200 mM NaCl, 0.02% NaN_3_ and stained with methylamine tungstate (Nano-W®, Nanoprobes). Images were acquired using an FEI Talos operated at 200 kV, equipped with an FEI Ceta 16M camera (4 k × 4 k). Image processing and 2D classifications were carried out using cisTEM^63^. Initial classification was performed to remove junk particles, followed by averaging into 15 (PRDM-CR9501), 10 (PRDM-motavizumab), or 10 (PRDM-CR9501-motavizumab) classes.

### Surface plasmon resonance competition assay

Biacore X100 (GE Healthcare) was used to measure the binding of CR9501 Fab and motavizumab Fab to trimeric prefusion F (both DS-Cav1 and PR-DM variants) or monomeric prefusion F (DS-Cav1 variant). Proteins were captured using an NTA sensor chip, which was regenerated between each cycle using 0.35 M ethylenediaminetetraacetic acid (EDTA) followed by 0.5 mM NiCl_2_. For each variant, five different sets of injections were performed using 200 nM solution for all Fabs and HBS-P+ pH 8.0 for buffer: (1) buffer, buffer, buffer; (2) buffer, buffer, CR9501; (3) buffer, buffer, motavizumab; (4) buffer, motavizumab, CR9501; (5) buffer, CR9501, motavizumab. The data were double-reference subtracted using the buffer-only reference samples.

### Cell-surface expression of F and flow cytometric analysis

Vectors encoding full-length wild-type RSV F variants, variants containing the DS-Cav1 or PR-DM stabilizing mutations, or a variant in which the cytoplasmic tail was replaced with a Foldon trimerization motif were cotransfected with a plasmid encoding furin in FreeStyle 293-F cells (ThermoFisher Scientific, Cat. No. R79007) or HeLa cells (ATCC-CCL-2). After approximately 24 h, cells were washed with blocking buffer (PBS with 0.5% bovine serum albumin (BSA), 1 mM EDTA), and incubated with a 500 nM solution of CR9501 Fab or motavizumab Fab for 30 min. For HeLa cells, adherent cells were stained on the surface of 48-well plates using PBS with 0.5% BSA. Solutions containing 1 mg mL^−1^ of motavizumab IgG or CR9501 IgG in 2 mM Tris pH 8.0, 200 mM NaCl and 0.02% NaN_3_ were fluorescently labeled using the Mix-n-Stain^TM^ CF®647 antibody labeling kit (Sigma) according to the manufacturer’s instructions and a 100 nM solution of labeled IgGs was added to the cells and incubated for 20 min. HeLa cells were then removed from 48-well plates using PBS with 0.5% BSA and 5 mM EDTA. Cells were resuspended in a solution containing a 1:2000 dilution of SYTOX Blue Dead Cell Stain (Molecular Probes) before analysis using a MACSQuant (Miltenyi) or a LSRFortessa SORP Flow Cytometer (BD). Due to lower levels of F protein expression in HeLa cells, these cells were gated on positive CF-647 signal before analysis. Experiments shown are the result of duplicate measurements for three biologically independent experiments.

For monoclonal antibody binding, a 500 nM solution of motavizumab, CR9501, AM22, AM14 or ADI-15576 IgG in blocking buffer was added to cells and incubated for 30 min. Cells were washed and a 1:300 dilution of mouse anti-human Fc-PE (SouthernBiotech, Cat. No. 9040-09) was added and incubated for 20 min. Cells were resuspended in a solution containing a 1:2000 dilution of SYTOX Blue Dead Cell Stain (Molecular Probes) before analysis using a LSRFortessa SORP Flow Cytometer (BD).

### Surface plasmon resonance affinity measurements

Trimeric or monomeric DS-Cav1, each with a C-terminal His tag, were immobilized on an NTA sensor chip to approximately 175 or 150 response units (RU), respectively, per cycle using a Biacore X100 (GE Healthcare). The sensor chip was regenerated between cycles using 0.25 M EDTA followed by 0.5 mM NiCl_2_. A buffer-only sample was injected over the sample and reference flow cells, followed by CR9501 Fab threefold serially diluted from 100 to 0.4 nM in HBS-P+, with a duplication of the 3.7 nM concentration. The data were double-reference subtracted and fit to a 1:1 binding model using Scrubber2.

### Reporting summary

Further information on research design is available in the Nature Research Reporting Summary linked to this article.

## Supplementary information

Supplementary Information

Reporting Summary

Description of Additional Supplementary Files

Supplementary Movie 1

Supplementary Movie 2

Supplementary Movie 3

## Data Availability

Atomic coordinates and structure factors for the monomeric prefusion RSV F-CR9501 Fab structure and the ternary prefusion RSV F-CR9501 Fab-motavizumab Fab structure have been deposited with the Protein Data Bank under accession codes 6OE4 and 6OE5, respectively. The authors declare that all other data supporting the findings of this study are available within the article and its Supplementary Information files, or are available from the authors upon request.
