# Supplementary material for: Transient opening of trimeric prefusion RSV F proteins
Source: Nat Commun. 2019 May 8;10:2105. doi: 10.1038/s41467-019-09807-5 (PMC6506550; doi:10.1038/s41467-019-09807-5)
Supplement: Supplementary file 1 — Supplementary Information [file 41467_2019_9807_MOESM1_ESM.pdf]

Supplementary Information for

Transient Opening of Trimeric Prefusion RSV F Proteins

Authors: Morgan S.A. Gilman[1,2], Polina Furmanova-Hollenstein[3], Gabriel Pascual[4], Angélique B. van ‘t Wout[5,6], Johannes P. M. Langedijk[3*], Jason S. McLellan[2*]

Affiliations:

[1]Department of Biochemistry and Cell Biology, Geisel School of Medicine at Dartmouth, Hanover, New Hampshire, USA 03755

[2]Department of Molecular Biosciences, University of Texas at Austin, Austin, Texas, USA 78712

[3]Janssen Infectious Diseases and Vaccines, Leiden, The Netherlands 2333 CN

[4]Janssen Immunosciences, World Without Disease Accelerator, San Diego, CA, USA 92121

[5]Janssen Prevention Center, Janssen Vaccines & Prevention B.V., Leiden, The Netherlands 2333 CN

[6]Current address: AlphaBiomics, London, United Kingdom SW4 0PA

*Correspondence: hlangedi@its.jnj.com; jmclellan@austin.utexas.edu

ethylenediaminetetraacetic acid

Supplementary Figures 1–9

Supplementary Tables 1–2

SUPPLEMENTARY FIGURES

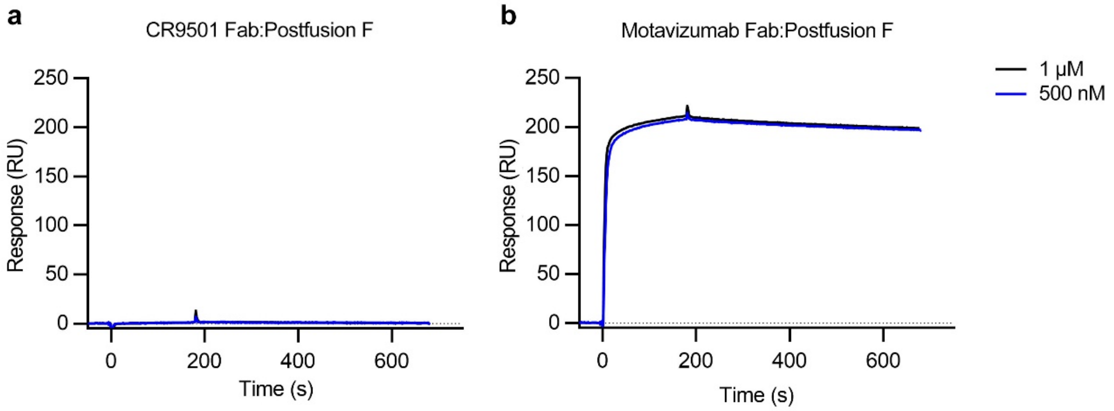

Supplementary Fig. 1. CR9501 does not bind to postfusion RSV F. Postfusion RSV F was captured on an NTA chip and binding of (a) CR9501 Fab or (b) motavizumab Fab was measured at 500 nM (blue) and 1 μM (black) concentrations of Fab using a Biacore X100.

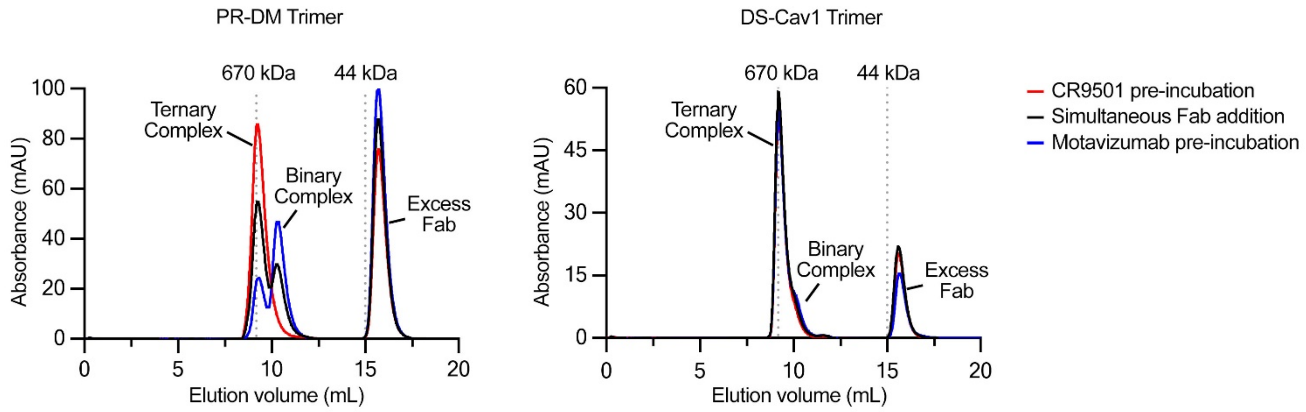

Supplementary Fig. 2. Competition between CR9501 and motavizumab for binding to prefusion F depends on the order of Fab addition and the nature of stabilizing mutations. Gel-filtration traces for complexes of PR-DM (left) and DS-Cav1 (right) formed by pre-incubation of CR9501 with prefusion F before addition of motavizumab Fab (red), simultaneous addition of motavizumab and CR9501 Fabs (black), or pre-incubation of motavizumab with prefusion F before addition of CR9501 Fab (blue).

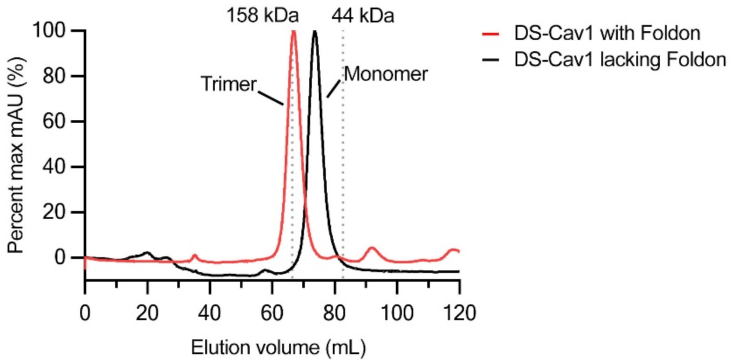

Supplementary Fig. 3. DS-Cav1 lacking the Foldon domain is monomeric. Gel filtration traces for DS-Cav1 either fused to a Foldon trimerization domain (red) or lacking this domain (black) measured using a Superdex 200 column (GE Healthcare) verify that DS-Cav1 without Foldon is monomeric.

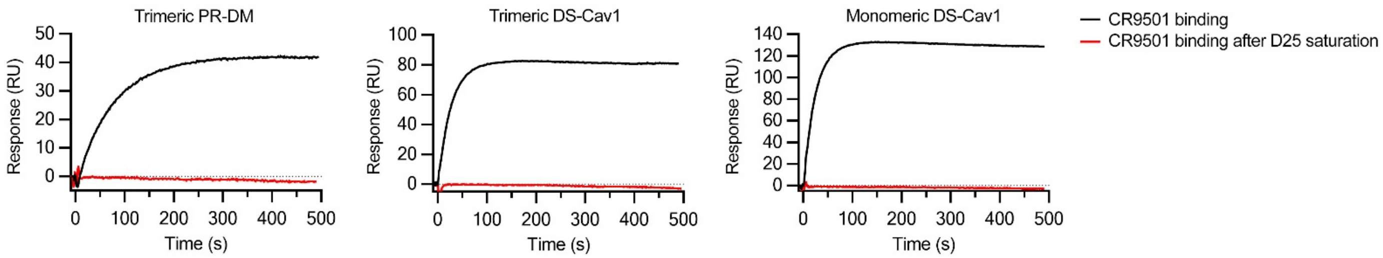

Supplementary Fig. 4. CR9501 and D25 compete for binding to monomeric and trimeric prefusion F. Two trimeric prefusion F variants (PR-DM or DS-Cav1) or monomeric prefusion F (monomeric DS-Cav1) were immobilized and saturating levels of D25 Fab (red) or buffer (black) was injected. CR9501 was then injected over the surface while measuring the binding response. D25 blocked binding of CR9501 to all three variants.

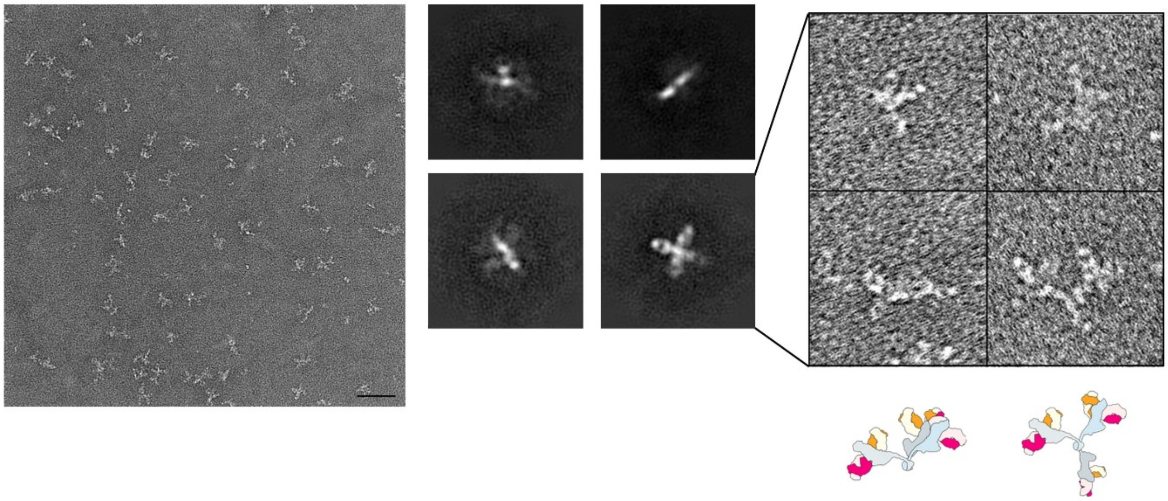

Supplementary Fig. 5. Splayed-open trimers are conformationally heterogeneous. Negative-stain EM of the prefusion F–CR9501–motavizumab complex formed by pre-incubation of PR-DM with motavizumab prior to addition of CR9501 (left, scale bar = 50 nm). Three representative class averages show uninterpretable views, whereas the fourth comprised individual monomers within splayed-open trimers bound by two Fabs (center). The inset shows a subset of the splayed-open trimers used to generate the fourth class average (right) and the cartoons illustrate the orientation of the ternary complex thought to be present in two of the particles.

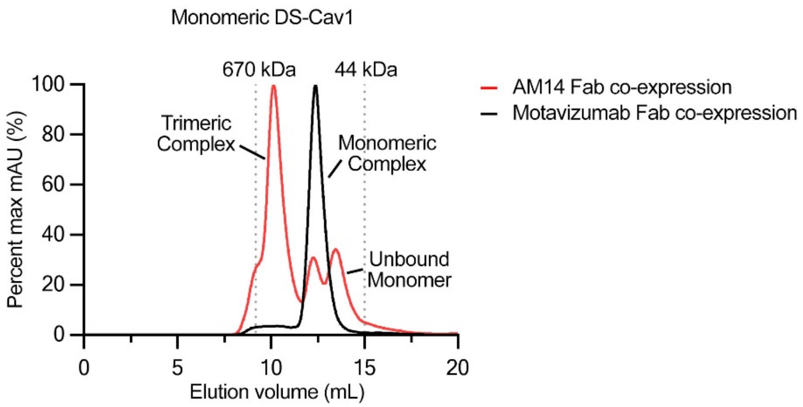

Supplementary Fig. 6. Motavizumab does not stabilize the prefusion F trimer. Gel filtration traces for monomeric DS-Cav1 co-expressed with motavizumab (black) or AM14 (red). Co-expression with AM14 resulted in stabilization of the prefusion F trimer whereas co-expression with motavizumab resulted in a monomeric complex.

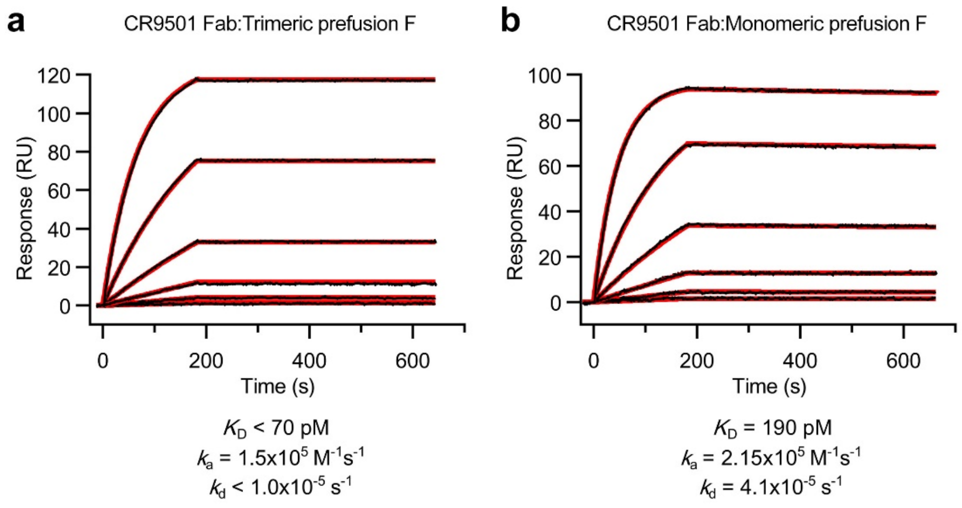

Supplementary Fig. 7. CR9501 Fab binds with high affinity to both trimeric and monomeric prefusion F. Binding of CR9501 to (a) trimeric and (b) monomeric prefusion F variants was measured using surface plasmon resonance. The data were double-reference subtracted (black) and fit to a 1:1 binding model (red).

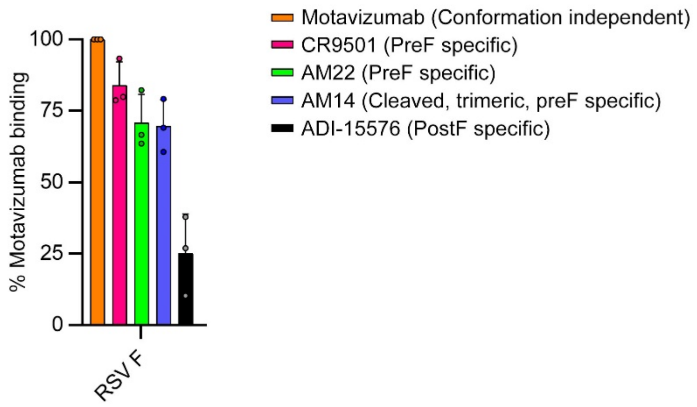

Supplementary Fig. 8. The quaternary-specific antibody AM14 binds RSV F at the cell surface. Binding of monoclonal antibodies that recognize both the pre- and postfusion conformations of F (motavizumab), only prefusion F (CR9501 and AM22), only cleaved, trimeric, prefusion F (AM14), or only postfusion F (ADI-15576) was measured by flow cytometry. Binding is expressed as percent of motavizumab binding to account for differences in expression levels. Results shown are derived from three biologically independent experiments.

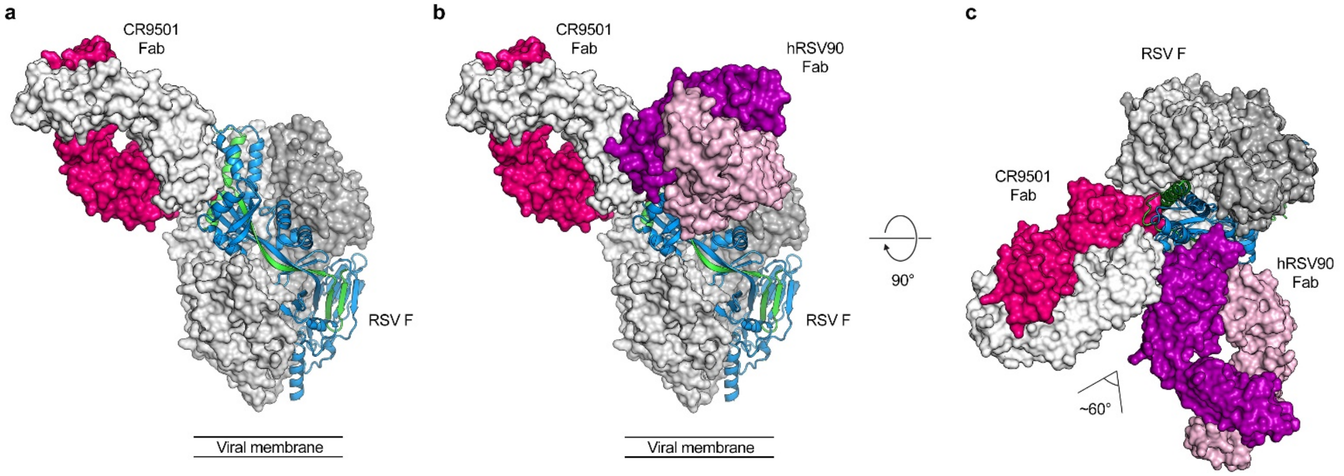

Supplementary Fig. 9. CR9501 and hRSV90 recognize antigenic site V with different angles of approach. (a) Monomeric prefusion F is shown as ribbons, with the F_1 subunit colored blue and the F_2 subunit colored green. The CR9501 heavy and light chains are shown as pink and white molecular surfaces, respectively. The two additional protomers of the prefusion F trimer are generated using the C3 symmetry observed in previous crystal structures and shown as molecular surfaces. (b) hRSV90 heavy and light chains are shown as dark and light purple molecular surfaces, respectively, and modeled binding to the same protomer as CR9501, colored as in (a). (c) Same as shown in (b) but rotated by 90° to show the trimer as viewed looking towards the viral membrane, highlighting the ~60° difference in the binding angles of CR9501 and hRSV90.

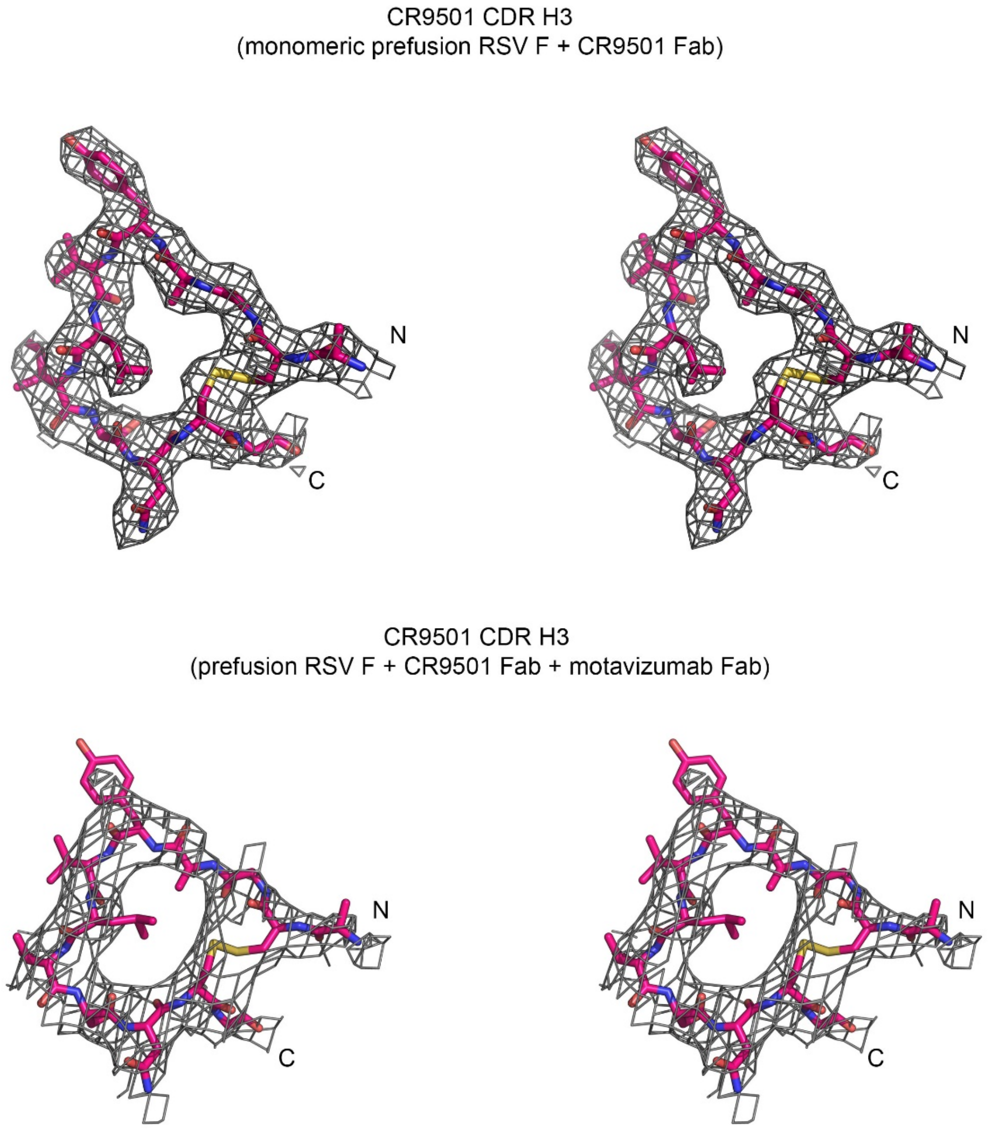

Supplementary Fig. 10. Electron-density maps for the CR9501 CDR H3. Stereo view of the refined 2*F*o - *F*c map generated in PyMOL and contoured at 1σ for the CDR H3 of CR9501 is shown for (top) the 3.3 Å structure of monomeric prefusion F bound to CR9501 Fab and (bottom) the 4.1 Å structure of prefusion F bound to CR9501 and motavizumab Fabs.

SUPPLEMENTARY TABLES

Supplementary Table 1. Shotgun mutagenesis suggests CR9501 binds near antigenic site V. Five variants identified by shotgun mutagenesis reduced CR9501 binding by more than 80% while retaining at least 35% binding to the control antibody (CR9502) and are colored to match Fig. 1.

Mutation	CR9501 (% WT)	Control (% WT)	Region on prefusion F
K168E	2.1	96.0	α3
L193A	9.1	46.5	β4
K156P	4.2	39.3	α2
N63H	5.2	57.2	F_2
Q94R	10.8	77.9	F_2

Supplementary Table 2. Crystallographic data collection and refinement statistics.

	Monomeric prefusion RSV F + CR9501 Fab	Prefusion RSV F + CR9501 Fab + motavizumab Fab
PDB ID	6OE4	6OE5
**Data collection**		
Space group	$P2_12_12_1$	$P6_1$
Cell dimensions		
a, b, *c* (Å)	105.6, 153.4, 155.5	219.8, 219.8, 68.8
α, β, γ (°)	90, 90, 90	90, 90, 120
Wavelength (Å)	0.9792	0.9793
Resolution (Å)	52.8–3.3 (3.45–3.30)	43.7–4.1 (4.58–4.10)
Unique reflections	38,582 (4,628)	15,219 (4,299)
R_{merge}	0.394 (1.757)	0.295 (1.267)
R_{pim}	0.173 (0.761)	0.116 (0.505)
I / σI	5.8 (2.2)	5.3 (1.7)
$CC_{1/2}$	0.963 (0.539)	0.988 (0.607)
Completeness (%)	99.7 (99.7)	99.9 (100.0)
Redundancy	6.1 (6.5)	7.3 (7.1)
Wilson *B*-factors (Å^2)	38.8	227.4
**Refinement**		
Resolution (Å)	52.8–3.3 (3.39–3.30)	43.7–4.1 (4.42–4.10)
Unique reflections	38,479 (2,906)	15,196 (3,004)
R_{work} / R_{free} (%)	21.2/25.9	26.2/30.6
No. atoms		
Protein	13,290	8,527
Ligand (EDO)	20	-
B-factors (Å^2)		
Protein	60.8	174.3
Ligand (EDO)	47.9	-
R.m.s. deviations		
Bond lengths (Å)	0.004	0.003
Bond angles (°)	0.87	0.50
Ramachandran		
Favored (%)	97.1	94.1
Allowed (%)	2.9	5.9
Outliers (%)	0	0

*Values in parentheses are for highest-resolution shell. One crystal was collected for each structure.

Supplementary Table 3. The apex of soluble, trimeric prefusion F adopts one of two distinct conformations.

PDB ID	Protein	pH	Space Group	Apex
4MMS	Cav1	5.5	$P4_12_12$	State 1
4MMU	DS-Cav1	5.5	$P4_132$	State 1
5K6B	DS-Cav1 9 sc	5.5	$P4_132$	State 1
5TPN	hRSV90-bound SC-TM	7.5	$H32$	State 1
5C69	PR DM	9.5	$P4_132$	State 1
5C6B	SC-TM	9.5	$P4_132$	State 1
5EA4	SM-bound DS-Cav1	9.5	$P4_132$	State 1
5EA8	DS-Cav1 D489Y	9.5	$P4_132$	State 1
5K6G	DS-Cav1 9-24 sc	5.5	$P4_132$	State 1
5TOJ	Nb4-bound DS-Cav1	4.5	$P3_221$	State 1
5TOK	NbL66-bound DS-Cav1	Untitrated	$P3_121$	State 1
5K6F	DS-Cav1 9-19 sc	5.6	$P4_132$	State 1
4ZYP	AM14-Mota-bound DS-Cav1	6.5	$P2_1$	State 1
5K6I	DS-Cav1 9-10 sc A149C-Y458C plus CR	6.5	$P4_132$	State 1
5U68	MPE8-scFv-bound DS-Cav1	7	$P3_121$	State 1
4JHW	D25-bound F	7.5	$P2_13$	State 1
5K6C	DS-Cav1 9-10 sc	7.5	$P4_132$	State 1
5EA5	SM-bound DS-Cav1	9.5	$P4_132$	State 1
5K6H	DS-Cav1 9-10 sc A149C-Y458C	4.2	$P4_132$	State 1
4MMT	DS-Cav1	9.5	$P4_132$	State 2
5EA6	SM-bound DS-Cav1	9.5	$P4_132$	State 2
5EA7	SM-bound DS-Cav1	9.5	$P4_132$	State 2
5EA3	SM-bound DS-Cav1	9.5	$P4_132$	State 2
4MMQ	DS	9.5	$P4_132$	State 2
4MMR	Cav1	9.5	$P4_132$	State 2
4MMV	DS-Cav1-TriC	9.5	$P4_132$	State 2

*SM = small-molecule; DS = disulfide bond; sc = single chain
