# Supplementary material for: Transient opening of trimeric prefusion RSV F proteins
Source: Nat Commun. 2019 May 8;10:2105. doi: 10.1038/s41467-019-09807-5 (PMC6506550; doi:10.1038/s41467-019-09807-5)
Supplement: Supplementary file 2 — Reporting Summary [file 41467_2019_9807_MOESM2_ESM.pdf]

nature research

Corresponding author(s): Jason S McLellan, Johannes PM Langedijk

Last updated by author(s): 03/27/2019

Reporting Summary

Nature Research wishes to improve the reproducibility of the work that we publish. This form provides structure for consistency and transparency in reporting. For further information on Nature Research policies, see Authors & Referees and the Editorial Policy Checklist.

Statistics

For all statistical analyses, confirm that the following items are present in the figure legend, table legend, main text, or Methods section.

n/a	Confirmed	
☐	☒	The exact sample size (*n*) for each experimental group/condition, given as a discrete number and unit of measurement
☐	☒	A statement on whether measurements were taken from distinct samples or whether the same sample was measured repeatedly
☒	☐	The statistical test(s) used AND whether they are one- or two-sided<br>*Only common tests should be described solely by name; describe more complex techniques in the Methods section.*
☒	☐	A description of all covariates tested
☒	☐	A description of any assumptions or corrections, such as tests of normality and adjustment for multiple comparisons
☒	☐	A full description of the statistical parameters including central tendency (e.g. means) or other basic estimates (e.g. regression coefficient) AND variation (e.g. standard deviation) or associated estimates of uncertainty (e.g. confidence intervals)
☒	☐	For null hypothesis testing, the test statistic (e.g. *F*, *t*, *r*) with confidence intervals, effect sizes, degrees of freedom and *P* value noted<br>*Give P values as exact values whenever suitable.*
☒	☐	For Bayesian analysis, information on the choice of priors and Markov chain Monte Carlo settings
☒	☐	For hierarchical and complex designs, identification of the appropriate level for tests and full reporting of outcomes
☒	☐	Estimates of effect sizes (e.g. Cohen's *d*, Pearson's *r*), indicating how they were calculated

Our web collection on statistics for biologists contains articles on many of the points above.

Software and code

Policy information about availability of computer code

Data collection	Biacore X100 Control Software 2.0.1 (GE Healthcare), FACSDiva 6.1.3 (BD), Tecnai Imaging & Analysis Software, SBCCOLLECT, Biotek Powerwave HT 340
Data analysis	Biacore X100 Evaluation Software 2.0.1 (GE Healthcare), FlowJo 10.5.2, Flowing Software 2.5.1, SPSS, cisTEM, SBGrid, CCP4i 7.0.021 and 6.5.019, iMOSFLM 7.2.1, AIMLESS 0.5.28 and 0.5.26, PHASER 2.6.1 and 2.5.7, PHENIX 1.12 and 1.13, Coot, GraphPad Prism 7.04

For manuscripts utilizing custom algorithms or software that are central to the research but not yet described in published literature, software must be made available to editors/reviewers. We strongly encourage code deposition in a community repository (e.g. GitHub). See the Nature Research guidelines for submitting code & software for further information.

Data

Policy information about availability of data

All manuscripts must include a data availability statement. This statement should provide the following information, where applicable:

- Accession codes, unique identifiers, or web links for publicly available datasets
- A list of figures that have associated raw data
- A description of any restrictions on data availability

Atomic coordinates and structure factors for the monomeric prefusion RSV F–CR9501 Fab 581 structure and the ternary prefusion RSV F–CR9501 Fab–motavizumab Fab structure have been deposited with the PDB under accession codes 6OE4 and 6OE5, respectively.

Field-specific reporting

Please select the one below that is the best fit for your research. If you are not sure, read the appropriate sections before making your selection.

☒ Life sciences ☐ Behavioural & social sciences ☐ Ecological, evolutionary & environmental sciences

For a reference copy of the document with all sections, see nature.com/documents/nr-reporting-summary-flat.pdf

Life sciences study design

All studies must disclose on these points even when the disclosure is negative.

Sample size	No sample size calculations were performed.
Data exclusions	No data were excluded.
Replication	All attempts at replication were successful.
Randomization	Samples were not randomized in these studies.
Blinding	Blinding was not used in these studies.

Reporting for specific materials, systems and methods

We require information from authors about some types of materials, experimental systems and methods used in many studies. Here, indicate whether each material, system or method listed is relevant to your study. If you are not sure if a list item applies to your research, read the appropriate section before selecting a response.

Materials & experimental systems

n/a	Involved in the study	
☐	☒	Antibodies
☐	☒	Eukaryotic cell lines
☒	☐	Palaeontology
☒	☐	Animals and other organisms
☒	☐	Human research participants
☒	☐	Clinical data

Methods

n/a	Involved in the study	
☒	☐	ChIP-seq
☐	☒	Flow cytometry
☒	☐	MRI-based neuroimaging

Antibodies

Antibodies used	mouse anti-human Fc-PE (SouthernBiotech, Cat No 9040-09), CR9502, CR9501, Motavizumab, AM14, AM22, ADI-15576, Alexa Fluor 488-conjugated secondary antibody (Jackson ImmunoResearch Laboratories)
Validation	Secondary antibody specificity: Human/Rhesus/Chimpanzee IgG Fc. Quality tested for flow cytometry. CR9501 and CR9502 were previously described (Krarup, A. et al. A highly stable prefusion RSV F vaccine derived from structural analysis of the fusion mechanism. Nat Commun 6, 8143 (2015).). Motavizumab binding to site II was structurally characterized previously (McLellan et al., Structural basis of respiratory syncytial virus neutralization by motavizumab Nat Struct Mol Biol 17(2): 248-250 (2011).). AM14 was previously characterized (Gilman, M.S. et al. Characterization of a Prefusion-Specific Antibody That Recognizes a Quaternary, Cleavage-Dependent Epitope on the RSV Fusion Glycoprotein. PLoS Pathog 11, e1005035 (2015).). AM22 has been characterized previously (McLellan, J.S. et al. Structure of RSV fusion glycoprotein trimer bound to a prefusion-specific neutralizing antibody. Science 340, 1113-7 (2013).). ADI-15576 has also been described previously (Gilman, M.S. et al. Rapid profiling of RSV antibody repertoires from the memory B cells of naturally infected adult donors. Sci Immunol 1, aaj1879 (2016).).

Eukaryotic cell lines

Policy information about cell lines

Cell line source(s)	FreeStyle-293F cells (Invitrogen), HeLa cells (ATCC-CCL-2), Vero cells (ATCC-CCL-81), HEK293T cells
Authentication	Cell lines were purchased commercially and were not further validated.
Mycoplasma contamination	FreeStyle-293F cells (Invitrogen), HeLa cells (ATCC-CCL-2), Vero cells (ATCC-CCL-81) have tested negative for mycoplasma contamination, HEK293T cell assays were performed by Integral Molecular.

Commonly misidentified lines (See ICLAC register) | *Name any commonly misidentified cell lines used in the study and provide a rationale for their use.*

Flow Cytometry

Plots

Confirm that:

☐ The axis labels state the marker and fluorochrome used (e.g. CD4-FITC).

☐ The axis scales are clearly visible. Include numbers along axes only for bottom left plot of group (a 'group' is an analysis of identical markers).

☐ All plots are contour plots with outliers or pseudocolor plots.

☐ A numerical value for number of cells or percentage (with statistics) is provided.

Methodology

Sample preparation	Only commercially purchased cell lines were utilized in flow cytometry experiments.
Instrument	Analysis only - BD LSRFortessa SORP Flow Cytometer
Software	Analysis only - FACSDiva v6.1.3, FlowJo, Flowing Software
Cell population abundance	Only FACS analysis was performed.
Gating strategy	Only FACS analysis was performed.

☐ Tick this box to confirm that a figure exemplifying the gating strategy is provided in the Supplementary Information.
