# Supplementary material for: Transient opening of trimeric prefusion RSV F proteins
Source: Nat Commun. 2019 May 8;10:2105. doi: 10.1038/s41467-019-09807-5 (PMC6506550; doi:10.1038/s41467-019-09807-5)
Supplement: Supplementary file 3 — Description of Additional Supplementary Files [file 41467_2019_9807_MOESM3_ESM.pdf]

Description of Additional Supplementary Files

File Name: Supplementary Movie 1

Description: **Prefusion F trimer opening is required for simultaneous CR9501 and motavizumab binding.** The prefusion F trimer is viewed looking toward the viral membrane, with each protomer shown as molecular surfaces colored blue, gray and white. Motavizumab and CR9501 Fabs are shown as molecular surfaces. CR9501 heavy and light chains are colored dark pink and light pink and the motavizumab Fab heavy and light chains are colored orange and tan, respectively. Movie shows a morph from the closed prefusion F trimer to an open state that alleviates the clash between the two antibodies.

File Name: Supplementary Movie 2

Description: **The apex of the prefusion F trimer exists in at least two states**. The prefusion F trimer is viewed looking toward the viral membrane, with each protomer shown as ribbons colored blue, gray and white. Movie shows a morph between the two prefusion F states, derived from two previously determined crystal structures (PDB IDs: 4MMU and 4MMT).

File Name: Supplementary Movie 3

Description: **Differences in the prefusion F apex are associated with motion in the α10 helix.** The prefusion F trimer is viewed looking along the viral membrane, with each protomer shown as ribbons colored blue, gray and white. Movie shows a morph between the two prefusion F states, derived from two previously determined crystal structures (PDB IDs: 4MMU and 4MMT).
